# Heroin-HIV-1 (H2) vaccine: induction of dual immunologic effects with a heroin hapten-conjugate and an HIV-1 envelope V2 peptide with liposomal lipid A as an adjuvant

**DOI:** 10.1038/s41541-017-0013-9

**Published:** 2017-05-02

**Authors:** Oscar B. Torres, Gary R. Matyas, Mangala Rao, Kristina K. Peachman, Rashmi Jalah, Zoltan Beck, Nelson L. Michael, Kenner C. Rice, Arthur E. Jacobson, Carl R. Alving

**Affiliations:** 1 US Military HIV Research Program, Henry M. Jackson Foundation for the Advancement of Military Medicine, 6720A Rockledge Drive, Bethesda, 20817 MD USA; 2 U.S. Military HIV Research Program, Walter Reed Army Institute of Research, 503 Robert Grant Avenue, Silver Spring, 20910 MD USA; 3 Department of Health and Human Services, Drug Design and Synthesis Section, Molecular Targets and Medications Discovery Branch, National Institute on Drug Abuse, National Institutes of Health, 9800 Medical Drive, Bethesda, 20892 MD USA; 4 National Institute on Alcohol Abuse and Alcoholism, National Institutes of Health, 9800 Medical Drive, Bethesda, 20892 MD USA

## Abstract

A synthetic heroin analog (MorHap) and a synthetic 42 amino acid V2 loop peptide from A/E strain of HIV-1 gp120 envelope protein that was previously used in a successful phase III vaccine trial were constructed as antigens together with liposomes containing monophosphoryl lipid A as an adjuvant, to explore the feasibility of producing a dual use vaccine both for treatment of heroin addiction and prevention of HIV-1 infection among injection drug users. The V2 peptide was tethered by a palmitoyl fatty acyl tail embedded in the liposomal lipid bilayer, and the heroin analog was conjugated to tetanus toxoid as a carrier protein that was mixed with the adjuvant. Upon comparison of a linear V2 peptide with a cyclic peptide, differences were found in the secondary configurations by circular dichroism, with the tethered cyclic peptide (palm-cyclic peptide) entirely in a random coil, and the tethered linear V2 peptide (palm-linear V2 peptide) entirely in a beta-sheet. Upon immunization of mice, palm-cyclic peptide induced anti-cyclic peptide endpoint titers >10^6^ and was considered to be a better immunogen overall than palm-linear V2 peptide for inducing antibodies to gp120 and gp70-V1V2. The antibodies also inhibited the binding of V2 peptide to the HIV-1 α_4_β_7_ integrin receptor. Antibody titers to MorHap, even with the presence of injected cyclic peptide, were very high, and resulted in inhibition of the hyper-locomotion and antinociception effects of injected heroin. From these initial experiments, we conclude that with a potent adjuvant and mostly synthetic constituents, a vaccine directed to heroin and HIV-1 (H2 vaccine) could be a feasible objective.

## Drug addiction: Dual-use vaccine shows promise for heroin and HIV

A vaccine designed to treat heroin addiction while at the same time preventing HIV infection elicited strong immune responses in mice. Scientists from the US government led by Carl Alving from the Walter Reed Army Institute of Research in Bethesda, Maryland, created a dual vaccine formulated with three main components: a segment of a protein expressed on the surface of HIV; synthetic molecules that resemble heroin and its degradation products; and a potent adjuvant to stimulate the immune system. Mice immunized with this vaccine had high antibody titers against the HIV surface protein as well as heroin and its derivatives. These mice also showed dulled responses to injected heroin. The findings suggest this vaccine strategy could help fight heroin abuse and the high risk of HIV infection among intravenous drug users.

## Introduction

Addiction to opioid drugs is a major source of morbidity and mortality worldwide, including the US, where it has become a national epidemic with an estimated 2.5 million adults afflicted in 2014.^1^ Of 44,000 drug-overdose deaths reported in the US in 2013, 19% (8360) were attributed to heroin, and 37% (16,280) were attributed to pharmaceutical opioids.^1^ In addition to overdose dangers, the sharing of needles represents a significant risk factor for infection with HIV-1.^2^ More than one-third of acquired immunodeficiency syndrome cases reported in the US have been attributed to injection drug use.^3, 4^ In the present work, we describe a strategy for formulating a combination vaccine for simultaneous use as a treatment modality for heroin addiction and as a candidate prophylactic vaccine for HIV-1 infection. The rationale behind this approach mirrors the strong association between injection drug use and the risk for HIV-1 infection, and it highlights the possibility of additive societal and health benefits of creating a single combination heroin-HIV-1 (H2) vaccine that has twin goals for alleviation of the two diseases.

Among the challenges for such a heroin-HIV vaccine are included individual antigen selection for the heroin and HIV-1 arms. In the case of heroin, rapid degradation to 6-acetyl morphine and morphine occurs after injection of heroin, and antibodies ideally are desired that would exhibit type 2 cross-reactivity both with heroin itself and with the major degradation products.^5, 6^ However, because of the extremely rapid degradation of heroin after injection, the bulk of euphoria is caused by 6-acetyl morphine and morphine, so although binding to heroin itself would be useful, it may not be absolutely necessary for achieving at least a high degree of vaccine efficacy.

For HIV-1, the exact immune response needed for efficacy and the optimal antigen required to achieve it are still unclear.^7^ To date, the only phase III clinical efficacy trial to demonstrate efficacy remains the RV144 Thai trial.^8^ The RV144 trial resulted in 31.2% efficacy for preventing HIV-1 infection, which, although modest, was significant at the primary endpoint of 3.5 years. However, post hoc analysis revealed apparent efficacies of 60 and 44%, respectively, at 12 and 18 months.^9^ Subsequent immune correlate analysis of RV144 revealed that non-neutralizing antibodies that were induced to the V1V2 loop of the HIV-1 gp120 envelope protein were inversely correlated with the risk of HIV-1 infection.^10^ In the gp120 protein, the V2 loop is located at the apex of the protein and has at least two α_4_β_7_ integrin-binding sites that play an important role in viral entry into susceptible cells, and could be important for vaccine design.^11–14^ The V1V2 region of gp120 has multiple N-linked glycan sites and the α_4_β_7_ integrin-binding site presumably could be obscured and protected by a “glycan shield”.^11–13^ As V2 is likely a relatively protected site, antibodies to gp120 injected as a vaccine antigen might be predominantly directed to sites, such as the V3 region, that are more immunodominant.^15^

**Fig. 1:**
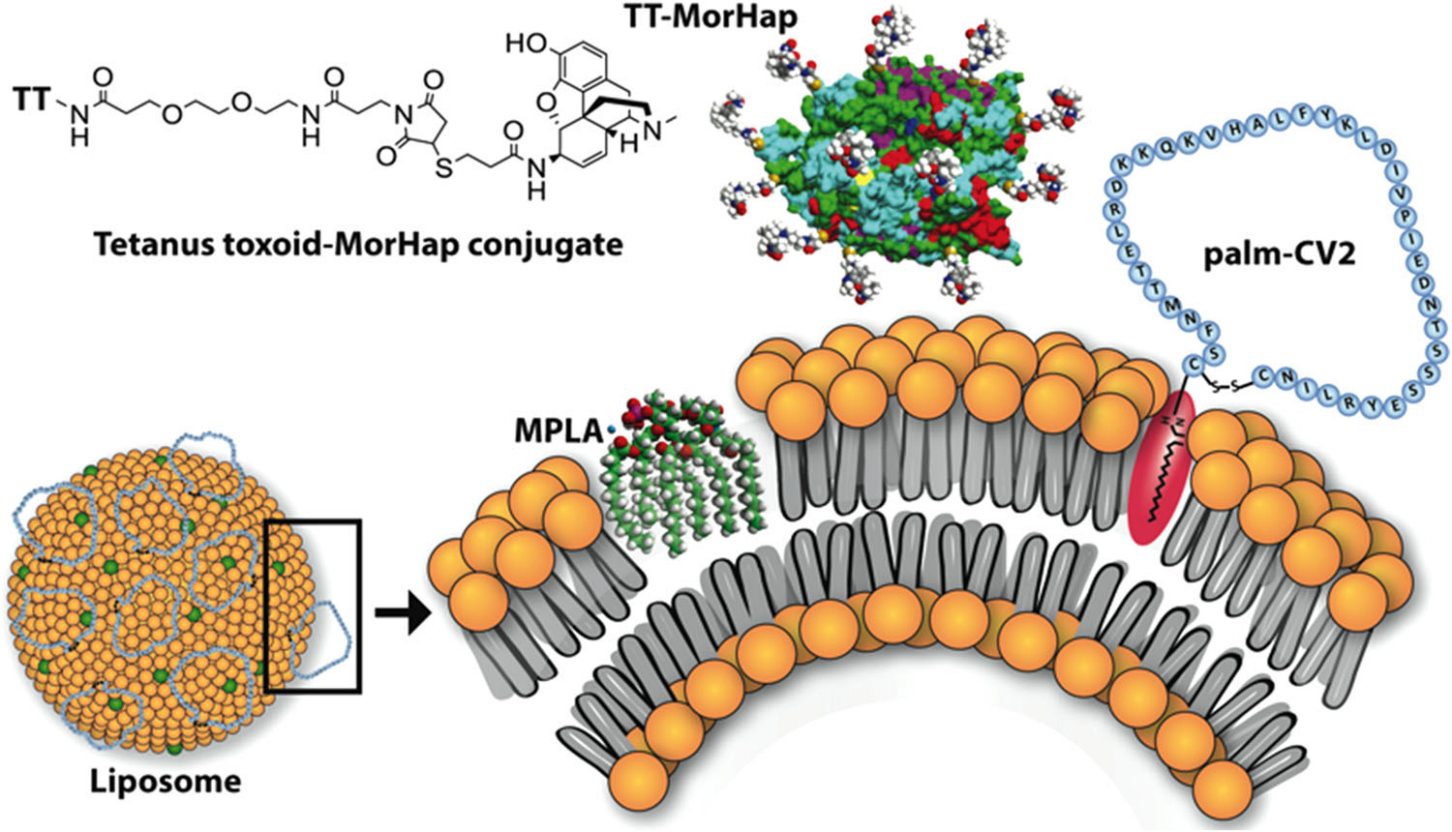
Schematic illustrations of combination vaccine formulations used for immunization of mice to induce antibodies to cyclic V2 peptide and MorHap. The palmitoyl moiety of palm-CV2 anchored the peptide to the lipid bilayer. MorHap (heroin hapten) was coupled to TT to yield TT-MorHap. The TT-MorHap and palm-CV2 was mixed with ALF, which served as an adjuvant. The entire TT was used for coupling with MorHap, but for simplicity the graphic illustrates the 2.3 Å X-ray crystal structure of tetanus neurotoxin light chain obtained from the RCSB Protein Data Bank

Based on the above considerations, in this study for the heroin arm, we included a synthetic hapten (MorHap) conjugated to tetanus toxoid that was predicted to display epitopes that would induce antibodies to heroin and particularly to its degradation products and inhibit the antinociception effects of heroin (Fig. 1).^5, 6, 16, 17^ For the HIV-1 arm, we examined two different antigens, consisting of either a synthetic linear (LV2) or a cyclic (CV2) 42 amino acid-free peptide, both of which were based on the V2 loop of an HIV-1 subtype AE (CRF01_AE) gp120 protein.^18^ The synthetic LV2 and CV2 peptides each contained palmitic acid at the N-terminus, resulting in palm-LV2 or palm-CV2. Antibodies to V2 from CRF01_AE HIV-1 in plasma samples obtained from the RV144 phase III Thai trial were previously detected by enzyme linked immunosorbent assay (ELISA) using linear and cyclic V2 peptides as capture antigens.^18^

For the adjuvant, the Army Liposome Formulation (ALF) adjuvant (liposomes containing monophosphoryl lipid A (MPLA)) was used as a potent adjuvant that would be expected to induce high levels of specific antibodies. The ALF family of adjuvants has been used in numerous phase I and II human trials since 1992 with various types of antigens, and has exhibited considerable safety and effectiveness. In previous studies, both in the development of a vaccine to heroin, and for induction of antibodies to peptides and proteins, ALF has shown a high level of potency as an adjuvant when compared with aluminum salts or many other adjuvants. ^19–22^ In this study, we incorporated either palm-LV2 or palm-CV2 as an immunogen into the surfaces of the ALF liposomal vaccine adjuvant particles, and we mixed the resultant particles together with the antigen consisting of MorHap conjugated to tetanus toxoid to make a multi-specific vaccine formulation (Fig. 1).

## Results

### Interactions of palm-CV2 and palm-LV2 with ALF adjuvant

**Fig. 2:**
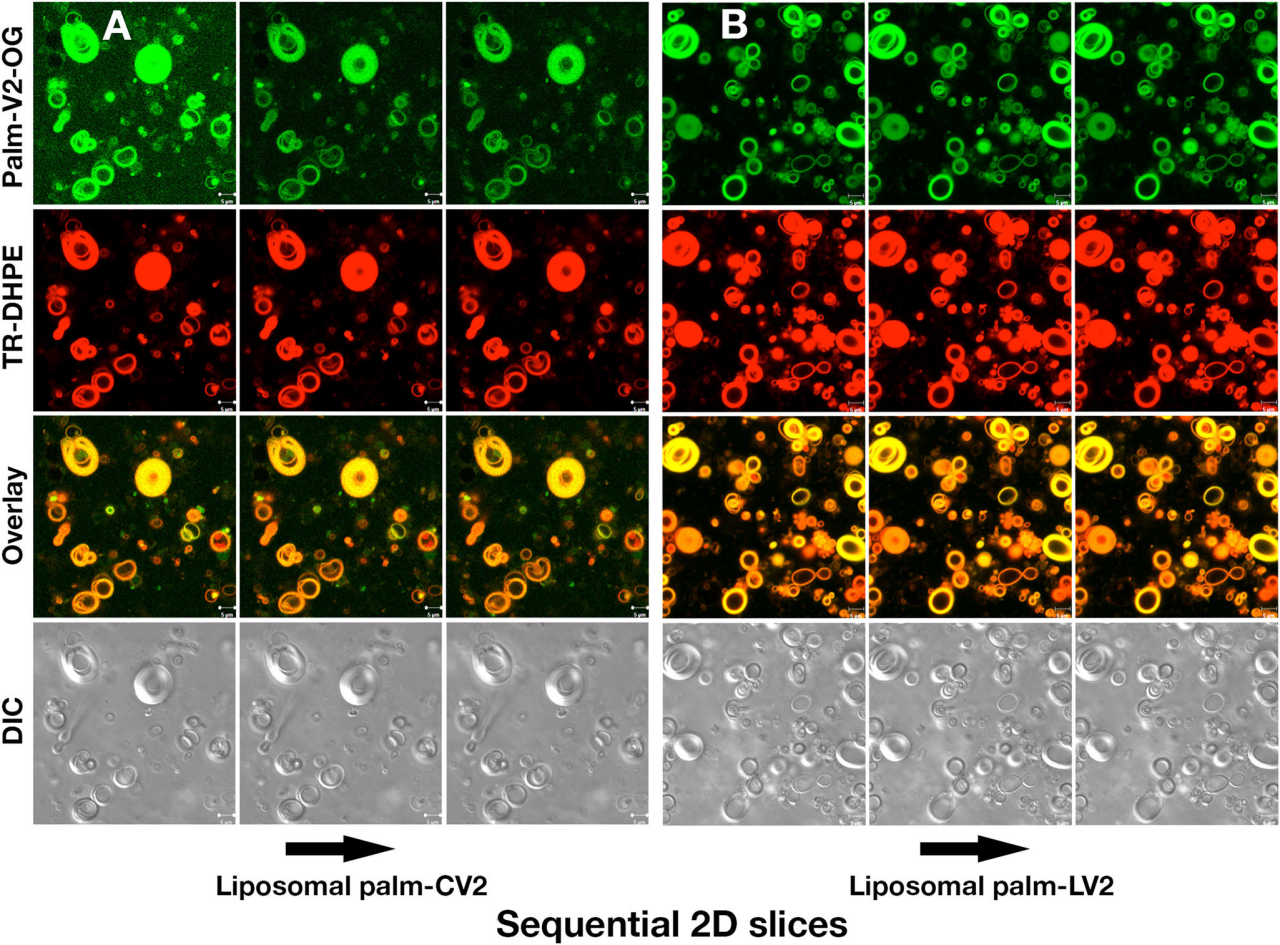
Confocal fluorescence microscopy images of multilamellar liposomes containing DHPE lipid labeled with Texas Red and palm-CV2-OG conjugate. The green and red fluorescence, and DIC channels for liposomal palm-CV2 (**a**) and liposomal palm-LV2 (**b**) are shown. The overlay of *green* and *red* channels is also shown. The 2D slices of the liposomes are represented in sequence (from *left* to *right*)

To visualize association of the V2 peptides with the ALF liposomal bilayer, palm-CV2 (Fig. 2a) or palm-LV2 peptide (Fig. 2b) was conjugated with Oregon Green (Palm-V2-OG) and the liposomes were labeled with Texas Red-DHPE phospholipid. The resultant formulations were subjected to confocal fluorescence microscopy (Fig. 2). As illustrated by three sequential 2-dimensional slices, together with overlay of the fluorescence of palm-V2-OG and TR-DHPE, the palm-CV2 and palm-LV2 each was associated entirely with the liposomes and the sequential slices, and overlay revealed no V2 (*green*) fluorescence outside the lipid bilayers (*red*).

**Fig. 3:**
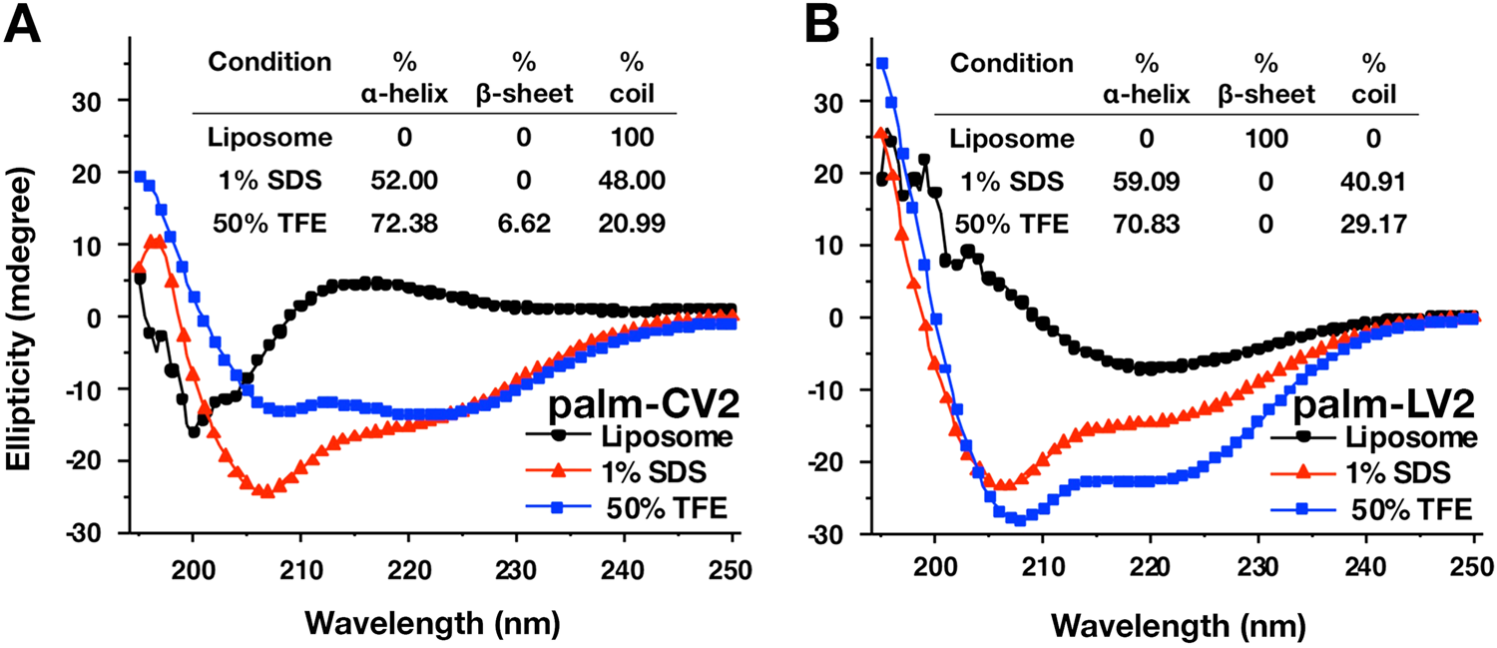
CD spectra. Palm-CV2 (3A) and palm-LV2 (3B) were examined under different experimental conditions. The traces represent an average of three scans

In order to determine the secondary structural configurations of the peptides, circular dichroism (CD) analysis was performed with liposomal palm-CV2 (Fig. 3a) or liposomal palm-LV2 (Fig. 3b), and with palm-CV2 or palm-LV2 in 1% sodium dodecyl sulfate (SDS) or in 50% trifluoroethanol (TFE). As shown in Fig. 3, the palm-CV2 and palm-LV2 peptides in TFE exhibited predominantly an alpha-helical structure (72%). Both of the constructs in SDS exhibited a mixture of alpha-helical and random coil. In contrast, the liposomal palm-CV2 peptide was entirely (100%) in a random coil. In addition, the liposomal palm-LV2 exhibited a beta sheet structure. Thus, it appears that association with the liposomal bilayers in ALF caused the palm-CV2 and palm-LV2 peptides to assume configurational structures that were different from each other and from those of non-liposomal CV2 and LV2 peptides.

### Immune responses of palm-CV2 in ALF compared with palm-LV2 peptide in ALF adjuvant

**Fig. 4:**
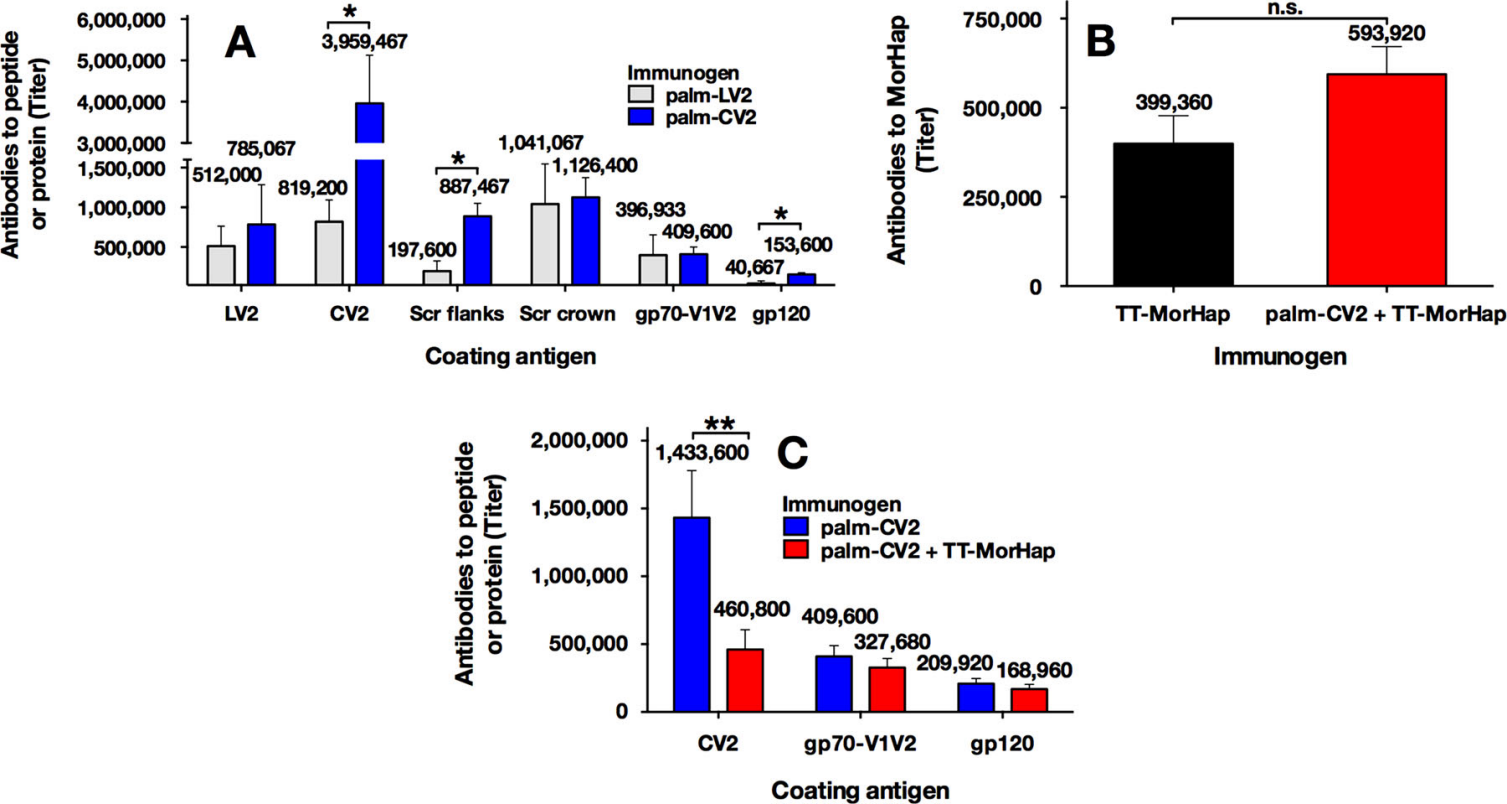
Serum IgG antibodies specific to peptides and MorHap. **a** Mice (six per formulation) were immunized with palm-LV2 or palm-CV2 and bled at week 9 and assayed for antibodies to LV2, CV2, scrambled (Scr) flanks, Scr crown, gp70-V1V2 and gp120. **b** Mice (ten per formulation) were immunized with TT-MorHap or palm-CV2+TT-MorHap, bled at week 8, and assayed for antibodies to MorHap. **c** Mice (ten per formulation) were immunized with palm-CV2 or palm-CV2+TT-MorHap, bled at week 8, and assayed for antibodies to CV2, gp70-V1V2, or gp120. Individual serum samples were analyzed by ELISA. Values are mean endpoint titers ± SEM. Asterisks indicate significant difference between the two vaccine formulations using a Mann–Whitney *t* test (*p < 0.05; **p < 0.01)

Immunization with liposomal palm-CV2 induced antibodies to CV2 with an endpoint titer of 3,959,467 (Fig. 4a). The anti-CV2 antibodies cross-reacted strongly with A244 gp70- V1V2 (titer 409,600) and gp120 (titer 153,600) (Fig. 4a). The anti-CV2 antibodies were non-neutralizing when tested with several tier 1 viruses (data not shown). To determine whether cyclization of the V2 was required for induction of high titers of antibodies, similar immunization was performed with palm-LV2. Titers of antibodies induced by palm-LV2 were not significantly different than those obtained by immunization with palm-CV2 either against LV2 (titers 512,000 and 785,067, respectively) or A244 gp70 V1/V2 (titers 396,933 and 409,000, respectively) (Fig. 4a). However, antibodies induced by liposomal palm-CV2 had significantly higher titers than those obtained by immunization with liposomal palm-LV2 for binding to CV2 peptide (titers 3,959,467 and 819,200, respectively) and to gp120 (titers153,600 and 40,667, respectively) (Fig. 4a). After immunization with either palm-CV2 or palm-LV2, the predominant IgG subtypes were IgG1 and Ig2a, with very little IgG2b or IgG3, thus suggesting a balanced Th1 and Th2 type response (Fig. S1).

In order to localize the specificities of induced anti-V2 peptide antibodies to regions of the intact V2 peptide, separate peptides were synthesized in which either the flank or the crown regions of intact V2 were scrambled (Scr flanks or Scr crown, respectively).^18^ In RV144, antibodies to V2 bound more strongly to Scr flanks peptides than to Scr crown peptide.^18^ In the present study, antibodies to palm-CV2 bound both to the Scr flank and Scr crown peptide. In contrast, antibodies to palm-LV2 bound predominantly to peptide containing Scr crown (titer 1,041,067) rather than to peptide containing Scr flank (titer 197,600). Based on these data, it was concluded both that palm-CV2 induced antibodies that bound to both the crown and flank regions of intact V2, and that palm-CV2 was a stronger immunogen than palm-LV2. Because of this, further experiments used liposomal palm-CV2 as an immunogen to determine the effect, if any, of simultaneous immunization with non-liposomal TT-MorHap as a second antigen.

### Anti-MorHap and anti-V2 immune responses of palm-CV2 with or without TT-MorHap

**Fig. 5:**
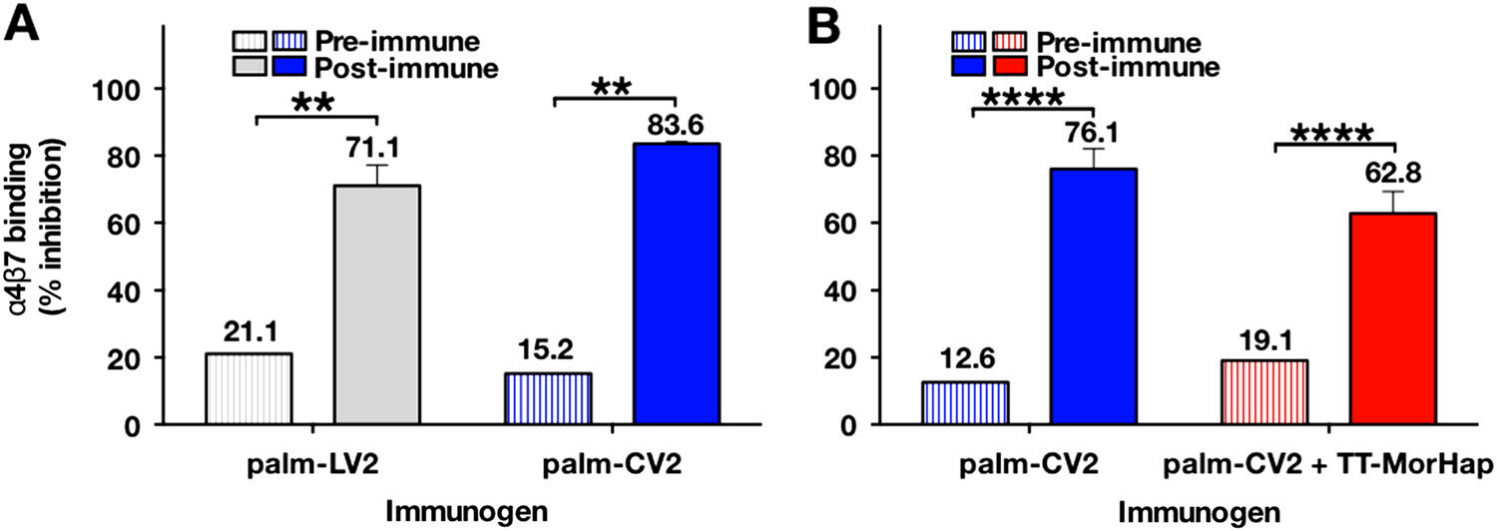
Inhibition of α4β7 binding to CV2 peptide by immune sera. **a** Mice (six per formulation) were immunized with palm-LV2 or palm-CV2 and bled at week 9. **b** Mice (ten per formulation) were immunized either with palm-CV2 or with palm-CV2+TT-MorHap, and were bled at week 15. The average % inhibition induced by immunization with palm-CV2 for the two animal studies was 80%. Values are mean ± SEM. Asterisks indicate significant difference between pre-immune and post-immune sera using a Mann–Whitney *t* test (**p < 0.01; ****p < 0.0001)

After immunizing with TT-MorHap (H vaccine), or with TT-MorHap together with palm-CV2 (H2 vaccine), high titers of antibodies were obtained to MorHap, but the titers of H and H2 vaccine to MorHap were not statistically different from each other (titers 399,360 and 593,920, respectively) (Fig. 4b). Although the H2 immunization, that included both palm-CV2 and TT-MorHap, resulted in significantly lower levels of antibodies to CV2 (titers 1,433,600 vs. 460,800, respectively), there were no significant differences in the cross-reactive antibody titers to A244 gp70-V1V2 (titers 409,600 and 327,680, respectively) or to gp120 (titers 209,920 and 168,960, respectively) (Fig. 4c). To determine the functionality of the antibodies, as shown in Fig. 5a, the antibodies induced either by palm-LV2 or palm-CV2 each strongly inhibited the binding of CV2 peptide to the α_4_β_7_ integrin receptor. Antibodies induced either by palm-CV2 or palm-CV2 plus TT-MorHap also strongly inhibited the α_4_β_7_ receptor (76.1 and 62.8% inhibition, respectively) (Fig. 5b).

### Inhibition of heroin-associated behavioral effects by antibodies induced by TT-MorHap with or without palm-CV2

**Fig. 6:**
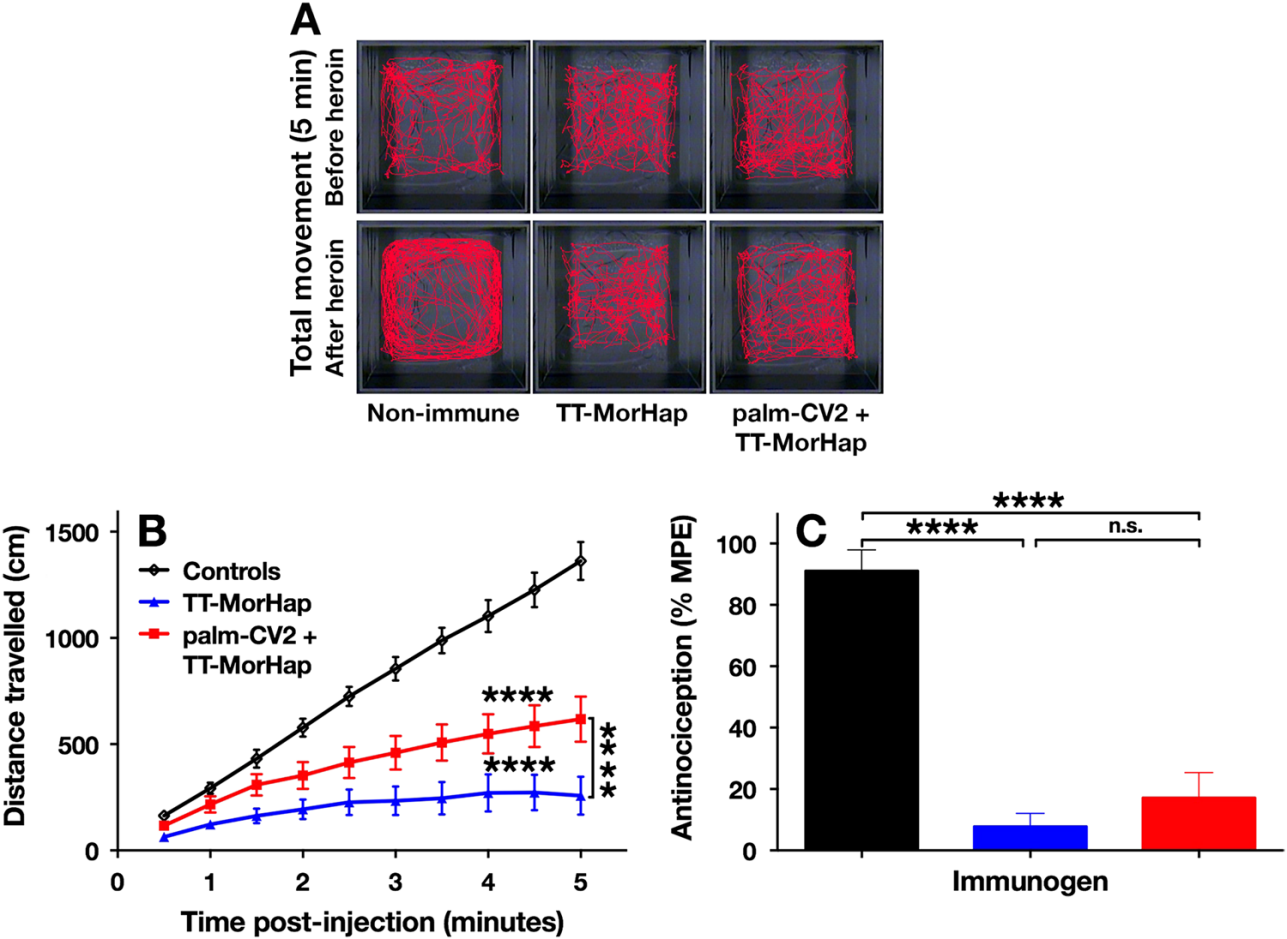
Heroin challenge of immunized and control mice. The locomotion (**a**) and distance travelled (**b**) by mice in 5 min were monitored before and after subcutaneous heroin injection. The *red* track is the visual tracing of the locomotion of a representative mouse. The distance travelled, which was derived from the track, is the difference in distance before and after heroin. The antinociception effect of heroin was measured by the tail flick test (**c**). Values are mean ± SEM. Asterisks indicate significant difference between immunized mice and controls using one-way ANOVA with Dunnett’s correction for multiple comparisons. (****p < 0.0001; n.s., not significant)

The biological effects of antibodies to heroin and related opioids were analyzed by inhibition of behavioral effects associated with injection of heroin. In the case of mice, injection of heroin is associated with increased hyper-locomotion and also by dulled reaction to pain (antinociception). Immunization with TT-MorHap either alone (H vaccine) or TT-MorHap together with palm-CV2 (H2 vaccine) caused significant reduction of heroin-associated hyper-locomotion (in Fig. 6a *middle* and *right bottom panels* are compared to the *control left bottom panel*). Inhibition of hyper-locomotion by each type of immunization was highly significant when compared to the controls (Fig. 6b). Inhibition of hyper-locomotion by antibodies to TT-MorHap alone was greater than inhibition by antibodies to TT-MorHap+palm-CV2. In addition, both types of immunization caused strong, highly significant, and equivalent inhibition of heroin-associated antinociception in immunized mice when compared to nonimmunized animals, as manifested by more rapid flicking of the tail that was exposed in a focused infrared light beam. (Fig. 6c). The presence of palm-CV2 as a simultaneous immunogen thus did not significantly change the inhibition of antinociception induced by TT-MorHap (Fig. 6c).

## Discussion

Heroin addiction and HIV-1 infection resulting from injection drug use can each have many disastrous personal health consequences for afflicted individuals, and they also represent complicated societal problems on many levels. To address these problems, we have created a dual vaccine formulation that explores the feasibility of immunotherapy of heroin-addicted persons and simultaneous prophylactic vaccination with an HIV-1 epitope to reduce further the chance of HIV-1 infection. The major initial challenges to achievement of these multifaceted goals are posed by the requirement for independent antigen selection for inducing immune responses both to heroin and to HIV-1. To simplify the complexity of potential antigenic structures, in this study, we used a combination of synthetic organic chemistry and conjugation chemistry for producing the heroin hapten conjugated to tetanus toxoid as a carrier protein, and peptide synthesis to focus on a relevant HIV-1 envelope peptide with a hydrophobic fatty acyl moiety that could be embedded in the liposomal lipid bilayer for display on the outer surface of liposomes. One of the goals of this initial work was to address the question of antigenic competition between a protein-conjugated heroin epitope and a synthetic HIV-1 peptide after immunization. Because of the simplified combination of individual small epitopes, in order to enhance the immunogenicity we have used a powerful synthetic adjuvant formulation, ALF (liposomes containing MPLA). This type of adjuvant has been successfully used previously for immunization with both heroin haptens and synthetic peptides.^19–22^ In previous work with MorHap conjugated to TT, using ALF as an adjuvant, we demonstrated that antibodies were induced that cross-reacted, with different levels of affinity, with heroin and its principal degradation products, 6-acetylmorphine and morphine.^16^ Although the affinities of the antibodies were higher for the degradation products of heroin than for heroin itself, because rapid degradation of heroin in blood 6-acetylmorphine and morphine are the major psychoactive compounds that result from heroin injection. In this study, we have demonstrated that although antigenic competition between the simple MorHap and V2 epitopes can sometimes occur, resulting in slightly lower titers, the ALF adjuvant provided stimulus for induction of very high end-point titers of antibodies both to MorHap and the V2 peptide in the combined vaccine.

The functionalities of the induced antibodies to heroin were also explored using two widely used measures of opioid-induced stimulation of μ-opioid receptors in mice, namely an antinociceptive effect (reduction of tail flick upon mild heat exposure),^23^ and hyper-locomotion after heroin injection.^24, 25^ In each of the two types of assay, highly significant inhibition was observed with mice immunized either with TT-MorHap alone or with TT-MorHap+CV2, when compared with non-immunized animals (Fig. 6). However, inhibition of heroin-induced hyper-locomotion was greater after immunization with TT-MorHap when compared to immunization with the combination of TT-MorHap and the CV2 peptide (Fig. 6b). In contrast, there was no significant difference between the inhibitory effects of the two antigenic formulations in the tail flick assay (Fig. 6c). The tail flick assay relies on a simple spinal reflex,^23^ while locomotion is “a complex behavior affected by many different brain systems, including [among others] the telencephalic dopaminergic system and the cerebellum…”.^26^ Although the reasons for the differences between the effects of antibodies induced by the two types of antigen formulations in the two assays is unknown, it could have reflected unknown subtle differences of affinities or other effects of the antibodies in the different assays. In each case, however, there were highly significant effects of each type of antigen formulation for inducing antibodies that reduced the functional effects of injected heroin.

For HIV-1, the CV2 peptide with a palmitoyl anchor in the surface of the ALF membrane induced antibodies that, both in the presence and in the absence of the heroin hapten-protein conjugate, resulted in strong inhibition of binding of HIV-1 to the α_4_β_7_ receptor that is present at the apex of the V2 loop of the HIV-1 gp120 envelope protein. The α_4_β_7_ receptor, which comprises at least two α_4_β_7_ integrin-binding sites,^14^ is closely associated with the CD4 HIV-1 binding site and is thought to play a role in HIV-1 infection.^27^ As noted in the Introduction, non-neutralizing antibodies to V1V2 were correlated with decreased risk of HIV-1 infection among vaccine recipients in the RV144 phase III trial,^10^ and this has brought attention to the potential importance of induction of antibodies to this region for vaccine design.^11–13^ Weakly neutralizing and non-neutralizing human monoclonal antibodies (mAbs) to the V2 loop were used as probes to study the interactions of anti-V2 antibodies with HIV-1, gp120 envelope protein and with the highly conserved V2 region, and its neighboring regions on gp120.^13, 15, 28^ Human anti-V2 mAbs derived from a volunteer vaccinee in RV144 bound both the free LV2 and CV2 peptides, including the conserved region of the α_4_β_7_ receptor, and inhibited the binding of HIV-1 to the receptor.^14, 29^ Anti-V2 mAbs derived from RV144 also captured a fraction, but not all, infectious HIV-1 virions from multiple HIV-1 strains in an in vitro capture assay.^29, 30^ Interestingly, combinations of different mAbs that bound to different regions of the HIV-1 gp160 envelope protein, such as mAbs binding both to V1/V2 on gp120 and to gp41, resulted in higher capture of infectious virions.^30^ This suggests that although antibodies to V2 might provide a useful degree of viral capture, future addition of multiple antigenic specificities to an H2 vaccine, such as addition of both V2 and gp41 epitopes, may result in the induction of antibodies that would provide better capture of infectious virions.

The potential importance of high titers of antibodies to V2, particularly with respect to the inhibition of binding of V2 to the α_4_β_7_ integrin receptor on CD4^+^T cells, as shown in this study, has been highlighted recently by the important observation that a monoclonal antibody to the α_4_β_7_ integrin receptor on SIV-infected cells, when combined with antiretroviral treatment, prevented rebound of suppressed SIV infection over a prolonged period, with no further treatment over the length of the study.^31^ The mechanism of this fascinating observation is currently unknown, but it would seem likely that inhibition or prevention by anti-α_4_β_7_ of initial homing of SIV to α_4_β_7_ on CD4^+^T cells in the mucosa was a major effect. Interestingly, the combined treatment of the infected macaques also resulted in increased titers of circulating antibodies to V2.^31^ Although the mechanism(s) for the increase in anti-V2 titers is unknown, it seems reasonable to assume that preservation of CD4^+^T cells from infection by SIV must have played a role. One could even speculate that blocking by anti-α_4_β_7_ antibodies of the binding of V2 of the SIV gp120 to α_4_β_7_ might have led somehow to increased exposure of the V2 binding site, thus allowing greater induction of anti-V2 antibody levels.

In the present study, distinctive secondary conformational differences based on CD analysis were observed between palm-CV2 and palm-LV2 upon association with liposomes, in that palm-CV2 was entirely in a random coil and the palm-LV2 was entirely in a *β*-sheet (Fig. 3). The relevance of secondary peptide conformation of V2, whether as *α* helix, *β* ribbon, or random coil, as it relates to induction of antibodies that inhibit the binding of V2 to α_4_β_7_ integrin receptor on susceptible cells is unknown. However, not all human mAbs that bind the V2 region have the capacity to inhibit the binding of V2 to the α_4_β_7_ integrin receptor,^14^ and the α_4_β_7_ receptor itself can also exhibit several different conformations that are in a dynamic flux.^32^ Regardless of the theoretical implications of differences of secondary structures of liposome-associated V2 peptides when compared with intact gp120 protein, in the present study immunization with liposomal CV2 and liposomal LV2 each exhibited strong inhibition of binding of CV2 to the cellular α_4_β_7_ integrin (Fig. 5). It should be pointed out that the inhibitions of binding of CV2 to α_4_β_7_ by the antisera in this study (62.8–83.6% inhibition) were greater than any of the inhibitions of binding of CV2 previously observed by us with human mAbs CH58, CH59, or 2158, or with individual plasmas obtained from infected patients in the RV144 study.^14^

The route to developing a maximal protective vaccine to HIV-1 is still not fully understood. The studies with mAbs suggest that anti-V2 loop antibodies may contribute at least partially to protective efficacy through a variety of mechanisms, possibly including inhibition of viral binding to α_4_β_7_ on CD4^+^T cells in the mucosa, ADCC induction by NK cells, or even through induction of neutralizing antibodies. In the present study, we have demonstrated that extremely high levels of antibodies to V2 that inhibit binding of HIV-1 to the α_4_β_7_ receptor can be achieved by direct attachment of the CV2 peptide to the highly potent ALF adjuvant. Thus, the feasibility and potential practicability has been demonstrated of combining a CV2 epitope together with a candidate heroin hapten along with a potent adjuvant formulation to achieve high levels of antibodies with small epitopes in a mostly synthetic H2 vaccine formulation. The resultant antibodies mitigated the effects of heroin injection and also exhibited high levels of functional antibodies to the V2 loop.

From a larger HIV-1 vaccine perspective, it might seem useful to include immunization with a V2 peptide as a complementary modality to induce high antibody titers to V2 that might contribute to protection, together with a protein antigen that might enlist additional protective mechanisms. Further studies will examine the addition of other haptens and peptides, and an HIV-1 protein to determine whether mixture with other antigenic epitopes could prove useful for additional additive or synergistic effects. However, in the present context, the H2 combination of a heroin vaccine with a simultaneous vaccine to the V2 loop of HIV-1 would treat the detrimental effects of injection drug use and might reduce the possibility of HIV-1 infection.

## Materials and methods

### Peptide synthesis

Peptides were synthesized on an Applied Biosystems 433 A peptide synthesizer using Fmoc chemistry. The two terminal cysteines were replaced with alanines to make the LV2 sequence.^18^ The resin was treated with either palmitic acid N-hydroxysuccinimide ester (for palmitoylated peptides) or biotin N-hydroxysuccinimide ester (for ELISA reagents) after the completion of the peptide synthesis. Palm-CV2, palm-LV2 and other peptides were cleaved from the resin using Reagent K and purified by reversed-phase high-performance liquid chromatography and characterized by MALDI-TOF MS. Palm-V2-OG conjugates were prepared using Oregon Green^®^ 488 (OG) carboxylic acid succinimidyl ester (Thermo Fisher Scientific, Waltham, Massachusetts, USA).

### TT-MorHap synthesis

TT-MorHap conjugate was synthesized, purified and characterized as previously described.^16^ TT-MorHap conjugates with 33–37 haptens were obtained by treating TT with 1600-fold excess, on a molar basis, of the heterobifunctional cross-linker.

### Vaccine formulation

ALF, consisting of dimyristoyl phosphatidylcholine: cholesterol:dimyristoyl phosphatidyglycerol (9:7.5:1 molar ratio), and synthetic MPLA (PHAD^®^; Avanti Polar Lipids, Alabaster, Alabama, USA) (400 μg/mL) were dissolved in lipid solvents as described earlier.^5^ To this were added either palm-CV2 or palm-LV2 peptide (400 μg/mL) in chloroform/methanol. The formulation for the HIV leg alone (50 μL dose) contained 50 mM phospholipid, 20 μg palm-V2 peptide and 20 μg MPLA in Dulbecco's phosphate buffered saline (DPBS), pH 7.4. For the heroin/HIV combination, preformed ALF was mixed 1:1 with the TT-MorHap conjugate. The heroin/HIV vaccine (50 μL dose) contained 10 μg of TT-MorHap, 20 μg palm-CV2 peptide, and the same amounts of phospholipids and MPLA as the HIV formulation.

### Immunization

All research in this study involving animals was conducted in compliance with the Animal Welfare Act, and other federal statutes and regulations relating to animals and experiments involving animals and adhered to the principles stated in the Guide for the Care and Use of Laboratory Animals, NRC Publication, 1996 edition. The research protocol was approved by the Institutional Animal Care and Use Committee of the Walter Reed Army Institute of Research and group sizes and animal numbers were selected in consultation with the statistician in the committee. Mice were randomly assigned to experimental groups and were not pre-screened or selected based on size or other gross physical characteristics.

Female Balb/c mice (6–7 weeks of age) were purchased from Jackson Laboratories (Bar Harbor, ME). Two sequential independent animal studies were conducted to assess the immunogenicity of the vaccine formulations: (1) comparison of palm-LV2 and palm-CV2 (6 mice/group), and (2) comparison of palm-CV2, TT-MorHap and palm-CV2+TT-MorHap (10 mice/group). Mice were immunized IM with 50 μL doses of the vaccines at weeks 0, 3, and 6 in alternating rear thighs. For the first animal study, six mice/group were terminally bled at week 9. For the second animal study, ten mice/group were bled at week 8, with a terminal bleed at week 15.

### Heroin challenge

The TT-MorHap and palm-CV2+TT-MorHap groups of mice were challenged with heroin (1 mg/kg, subcutaneous) at week 10.^16^ The estimated lethal dose of heroin in humans is 50 mg (range 12–180 mg).^33^ Assuming a 70 kg person, this corresponds to 0.7 mg/kg. The dose of injected heroin used in the current mouse studies is thus in-line with the toxic dose of heroin in humans.

### Confocal fluorescence microscopy

Liposomes were labeled with Texas Red-DHPE (0.25%, 12.5 μM) (Thermo Fisher Scientific) and the Palm-V2-OR peptides were added as 0.16% (8.0 μM). For confocal fluorescence microscopy, the total phospholipid concentration was 5 mM. Images were taken with a Zeiss LSM 710 confocal microscope (Zeiss, Oberkochen, Germany) equipped with excitation laser lines at 405, 458, 488, 514, 561, and 633 nm. Images were taken using a 63×/1.4 Oil DIC Plan Apo. The liposome-containing peptide formulations were placed in a CoverWell™ perfusion chamber gasket (Thermo Fisher Scientific), and palm-CV2 and palm-LV2 peptides and Texas Red-DHPE were observed with fluorescein and rhodamine filters, respectively. All images were stored and processed with the Zeiss Zen 2012 Lite software. Green fluorescent, red fluorescent, and gray images are Oregon Green, Texas Red and DIC channels, respectively. Superimposition of the Oregon Green and Texas Red channels yield the overlay images.

### Circular dichroism

CD experiments were performed under three conditions: liposomes (small unilamellar vesicles containing 2 mM phospholipid suspended in DPBS); 1% SDS in DPBS; and 50% TFE in DPBS (1%). The experiments were performed using 400 μg/mL of liposomal peptide, and 50 μM peptide in 1% SDS or 50% TFE, at 25 °C in a Hellma quartz cell (1 mm path length). Data points were recorded between 195 and 250 nm in 0.5 nm increments on a JASCO J-815 CD Spectrometer (Easton, Maryland, USA). Spectra were collected using scan speed of 10 nm/min and a band-pass of 1 nm. For each peptide condition assayed, three scans were recorded, averaged, and corrected for appropriate blanks. For CD of palm-V2 peptides in liposomes, the CD data of control empty liposomes was analyzed at 2 mM liposomal phospholipid concentration and subtracted from the sample data. The % secondary structure of the peptide was calculated by fitting the CD data through CDFIT Program.^34^

### Enzyme linked immunosorbent assay

MorHap ELISA was performed with 100 uL (1 μg/mL) of coating antigen.^17^ V2, A244 gp70-V1V2, and gp120 ELISAs were performed as described previously,^18^ with modifications, as summarized in Table S1).

### α_4_β_7_ Assay

Serum samples pre-immunization (pooled) or post-immunization (individual) at a dilution of 1:50 in sample buffer was examined as previously described.^14^ Sera from animals immunized with palm-CV2 and palm-LV2 were evaluated at week 9, while sera from animals immunized with palm-CV2 together with TT-MorHap were evaluated at week 15, for inhibition of binding of CV2 peptide to α_4_β_7_ on RPMI 8866 cells.

### Tail flick and locomotion tests

Mice were challenged with heroin at week 10. Tail flick test was performed as previously described.^16^ For the locomotion test, the same mice from the tail flick test were placed in a black chamber (11 inches × 11 inches) with an EverFocus^®^ Polestar II camera (EverFocus, Duarte, California, USA) mounted above (56 inches from the chamber), for 5 min. The locomotion of the mice was processed by EthoVision^®^ XT software (Noldus Information Technology Inc., Wageningen, The Netherlands). The baseline responses prior to heroin injection were measured on the tail flick followed by the locomotion test, after which the animals were injected SC with 1 mg/kg of heroin HCl in saline (400 μg/mL) between the front shoulders. Thirty minutes after injection, the tail flick responses were measured and the motions of the mice inside the box were tracked for 5 min. The distance of the tracks were recorded at 0.5 min interval for 5 min and the distance travelled at a given time was calculated using the formula:

$${\rm{Distance}}\,{\rm{travelled}}={{\rm{Distance}}}_{{\rm{after}}{\rm{heroin}}}-{{\rm{Distance}}}_{{\rm{before}}{\rm{heroin}}}$$

In each case, the distance travelled prior to heroin injection was subtracted as a baseline from the distance travelled after heroin injection.

## Electronic supplementary material

Supplementary information
