# Supplementary material for: Heroin-HIV-1 (H2) vaccine: induction of dual immunologic effects with a heroin hapten-conjugate and an HIV-1 envelope V2 peptide with liposomal lipid A as an adjuvant
Source: NPJ Vaccines. 2017 May 2;2:13. doi: 10.1038/s41541-017-0013-9 (PMC5604742; doi:10.1038/s41541-017-0013-9)
Supplement: Supplementary file 1 — Supplementary information [file 41541_2017_13_MOESM1_ESM.docx]

Heroin-HIV-1 (H2) vaccine: Induction of dual immunologic effects with

a heroin hapten-conjugate and an HIV-1 envelope V2 peptide

**with liposomal lipid A as an adjuvant**

Oscar B. Torres, Gary R. Matyas, Mangala Rao, Kristina K. Peachman,

Rashmi Jalah, Zoltan Beck, Nelson L. Michael, Kenner C. Rice,

Arthur E. Jacobson, Carl R. Alving

Supplementary Information

**Table S1.** Details of ELISA assays used for detecting antibodies to different antigens.

| ELISA type | ELISA  plate | Coating agent | Blocker | Incubation time | Sera incubation time | Washing buffer^c^ |
| --- | --- | --- | --- | --- | --- | --- |
| CV2 or LV2 | “U” bottom | Streptavidin^a^/  biotin-V2^b^ | 0.5% BSA, 0.5% casein | 2 hr, RT | Overnight, 4 °C | 1x DPBS, pH 7.4 |
| gp70-V1V2 | “U” bottom | gp70-V1V2^b^ | 0.5% milk | Overnight, 4 °C | 1 hr, RT | 1x DPBS,  pH 7.4 |
| gp120 | flat bottom | gp120^b^ | 0.5% milk | 2 hr, RT | 1 hr, RT | TBS,  pH 7.4 |
| ^a^ Overnight at 4 °C with streptavidin (200 ng) followed by biotin-V2 for 1 hr at 37 °C | | | | | | |
| ^b^ 100 ng of coating antigen | | | | | | |
| ^c^ with 0.1% Tween 20 | | | | | | |

**Figure S1.** IgG subclass profiles of anti-CV2 sera.


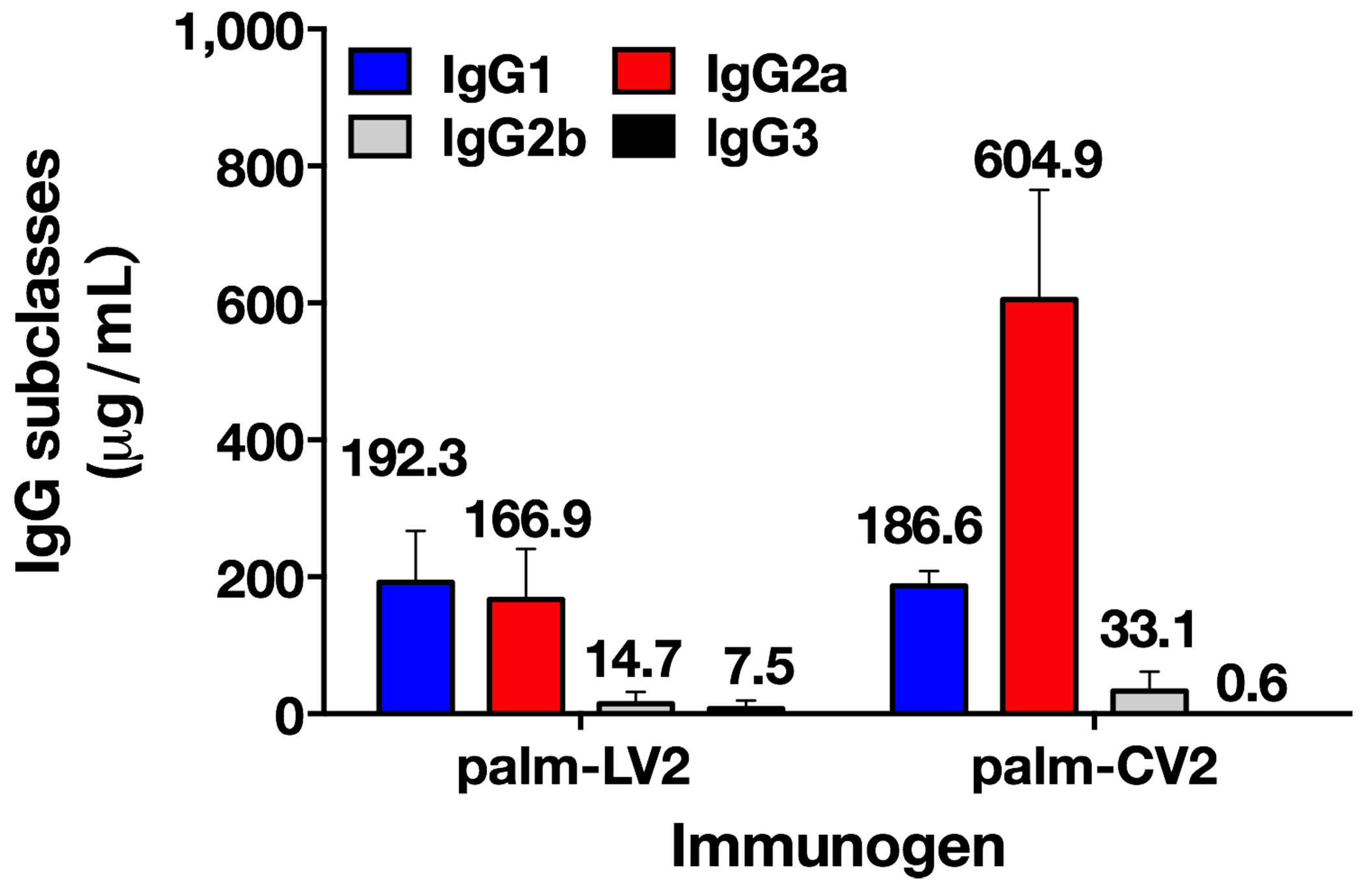


Mice (6 mice/formulation) were immunized with palm-LV2 or palm-CV2 and bled at week 9. Individual serum samples were analyzed by IgG subclass ELISA as described before.^1^ Values are mean IgG subclasses (μg/mL) ± SEM. Although wide variations were observed among the mice immunized with palm-CV2, there was no significant difference between the IgG1 and IgG2a concentrations using a Mann-Whitney t test.

1. Beck Z, Matyas GR, Jalah R, Rao M, Polonis VR, Alving CR. Differential immune responses to HIV-1 envelope protein induced by liposomal adjuvant formulations containing monophosphoryl lipid A with or without QS21. *Vaccine* **33**: 5578–5587 (2015)
